# Enhancing Musical Learning Through Mixed Reality: A Case Study Using PocketDrum and Meta Quest 3 for Drum Practice

**DOI:** 10.3390/s25226836

**Published:** 2025-11-08

**Authors:** Mariano Banquiero, Gracia Valdeolivas, M.-Carmen Juan

**Affiliations:** 1 Instituto Universitario de Automática e Informática Industrial, Universitat Politècnica de València, C/Camino de Vera s/n, 46022 Valencia, Spain; mbanqui@dsic.upv.es; 2 Centre D’estudis Musicals d’Onda, C/Ronda 20, 12200 Onda, Spain; gvaldeolivasn@conservatorionda.com

**Keywords:** mixed reality, virtual reality, immersive learning, hand tracking, music education, drum training, PocketDrum

## Abstract

This work presents a mixed reality application for drum learning that combines the PocketDrum virtual drumming device with the Meta Quest 3 headset, integrating hand tracking to provide an immersive, responsive experience without the need for a physical drum set. The system features a modular architecture for real-time strike detection, visual guidance synchronized with music, spatial calibration, and audio rendering. The system additionally makes use of the headset’s color Passthrough during the calibration stage to align the virtual drum kit with the player’s position. To evaluate the system’s performance, a technical analysis was conducted to measure latency, jitter, and sampling rate across the technologies involved. Additionally, a functional validation experiment assessed how spatial hand tracking from Meta Quest 3 improved PocketDrum’s classification accuracy. Results showed that the fused system corrected 19.1% of drum assignment errors made by the inertial-only setup, enhancing consistency in complex rhythmic patterns. These findings demonstrate the effectiveness of sensor fusion for immersive percussion training and support its potential use in accessible, feedback-rich musical learning environments.

## 1. Introduction

Drumming is a complex motor skill that requires precise timing, coordination, and spatial awareness. Traditionally, learning drums demands access to a physical drum set, which may be costly, noisy, or impractical in many contexts, particularly for beginners or students with space or budget constraints. As a result, alternative approaches such as electronic drums, mobile applications, and more recently, virtual reality (VR) and mixed reality (MR) systems have emerged to make drumming more accessible and versatile.

Among these, MR offers a unique opportunity to combine the immersive benefits of virtual environments with the contextual awareness of the real world. This opens the door to new interaction models that preserve key physical cues (e.g., holding drumsticks, foot placement) while enhancing feedback, visualization, and portability. However, creating a realistic and responsive MR drumming experience remains technically challenging due to issues such as input latency, spatial calibration, and sensor precision, especially in the absence of physical contact surfaces. Beyond the technical challenges, drumming also involves complex perceptual–motor learning processes. According to embodied and cognitive models of musical performance, rhythmic skill emerges from the tight coupling between perception, action, and mental imagery [1,2]. In this view, musicians continuously simulate and anticipate the sensory outcomes of their gestures, adjusting movement based on auditory and visual feedback. Preserving tactile interaction and providing synchronized multisensory cues, as achieved in our MR setup, supports this embodied loop of prediction and correction that underlies musical learning.

This work presents a MR application for drum learning that combines PocketDrum, a commercial device by Aeroband featuring motion-sensitive drumsticks and foot sensors, with the Meta Quest 3 headset and its hand tracking system. The proposed integration leverages the complementary strengths of both systems: high-frequency inertial strike detection and real-time spatial tracking. This hybrid architecture aims to deliver an immersive, accurate, and portable drumming experience without requiring physical drums.

To evaluate system performance, we conducted two complementary experiments. The first measured latency, jitter, and sampling rate in each subsystem to characterize their individual technical profiles. The second identified classification errors made by PocketDrum in realistic playing conditions and assessed how spatial tracking from Meta Quest 3 resolves these limitations.

The main contributions of this work are as follows:

- A modular MR architecture for virtual drumming that fuses fast inertial sensing with spatial hand tracking.
- A technical evaluation of latency, jitter, and sampling rate across the sensing technologies.
- A functional validation experiment that evaluates the limitations of PocketDrum when used alone and assesses how the integration of spatial hand tracking improves the reliability of the system.

Although no formal learning assessment was conducted in this study, the system is designed to support musical practice and rhythmic skill development, particularly for beginners or users with limited access to physical drum kits. By providing real-time visual and auditory feedback, and eliminating spatial and acoustic constraints, the proposed MR application has the potential to lower barriers to entry and facilitate more frequent, guided practice sessions. These aspects make it especially relevant for educational use cases, which will be explored in future user-centered studies.

This publication presents the system architecture and application design (Section 3), details two experimental evaluations, technical analysis and functional validation (Section 4 and Section 5), discusses the implications of the results (Section 6), and concludes with directions for future research (Section 7).

This work presents a mixed reality drumming system that integrates PocketDrum sensors with Meta Quest 3’s native hand tracking in a portable and wireless setup. The system combines sensor fusion, calibration, and synchronized audio–visual feedback, and is evaluated through both technical performance (latency, jitter, sampling rate) and functional classification accuracy.

## 2. Related Work

Several systems with different technologies have been proposed for musical learning, specifically for drumming [3,4]. In recent years, VR and MR have emerged as promising tools in this area, allowing users to practice without a physical drum set, often incorporating real-time visual or auditory feedback.

One of the earliest studies on virtual drumming was conducted by Kilteni et al. [5], who investigated how VR immersion affects musical performance by inducing body ownership illusions. Participants played a virtual hand drum in the first person while embodied in different avatars. The results showed that the perception of the virtual body influenced how participants played the instrument, establishing a link between body representation in VR and expressiveness in drumming.

A few years later, Ishiyama and Kitahara [6] developed a VR-based drum performance system aimed at remote ensemble playing. Their system allowed a drummer wearing a headset to see and interact with a virtual bassist whose movements were captured using Kinect. Their study explored how musicians could use non-verbal communication in a virtual setting to synchronize performances, demonstrating the feasibility of ensemble playing in VR.

In the field of educational applications, Kweon et al. [3] developed a drum learning system based on augmented reality (AR) and VR, designed to enhance students’ rhythmic accuracy and intuition. Their system provided visual guidance over a real drum set in AR or fully simulated drumming environments in VR. Users received real-time feedback through synchronized visual, auditory, and tactile cues, showcasing the potential of immersive learning for drumming.

More recently, Pinkl and Cohen [7] introduced an innovative approach using Virtual Co-Embodiment for drum training in VR. Their system incorporates a novel concept called a “Halvatar”: an avatar whose control is shared between the user and a pre-programmed animation. This configuration enables a co-performance scenario in which the learner interacts with a model-driven limb of the avatar. The system combines action observation techniques with real-time feedback to support the learning and execution of drum rudiments and polyrhythms in an immersive environment.

Building on this educational perspective, Wang et al. [8] introduced MR-Drum, an MR drumming system developed within the ICMI research community. Their approach integrates a structured micro-progression learning framework that decomposes rhythmic and limb-coordination tasks into gradual stages. A user study comparing MR-Drum with instructional videos showed significant improvements in timing accuracy and error reduction, underscoring the pedagogical value of MR environments for musical training.

Another recent approach is A2D (Anywhere Anytime Drumming), developed by Yadid et al. [9]. This vision-based drumming system employs computer vision and deep learning, eliminating the need for specialized hardware. Instead, users practice with generic drumsticks and a smartphone camera, which detects their movements and provides real-time auditory feedback. The use of neural networks and tracking filters enhances motion recognition accuracy, achieving near-perfect detection in user tests.

Inertial sensors have also been explored as an alternative to VR interfaces. Tavares et al. [10] developed a system using convolutional neural networks to predict drum hits in a virtual environment based on accelerometer data. Their study demonstrated how predictive models could enhance musical synchronization by identifying preparatory gestures and triggering sounds just before the actual impact, reducing perceived latency.

Pesek et al. [4] analyzed the effect of using a VR game as a complementary tool to improve users’ rhythmic performance and perception in a remote and self-learning environment. They developed a drum-playing game based on a tower defense scenario designed to enhance rhythmic perceptual skills in elementary school children. Over a 14-day individual training session with Meta Quest 2 headsets, participants showed significant improvement in their rhythmical skills, highlighting VR’s potential in music education.

Several drumming applications have emerged in the Meta Quest store in recent years, offering different approaches to virtual drumming. Some, like Smash Drums [11] and Drums Rock [12], focus on an arcade-style experience, where players follow rhythmic patterns to hit virtual drums in synchrony with the music. In both cases, a virtual drum set remains visible in front of the player, and incoming elements (such as symbols, icons or enemies) indicate when and where to strike. These cues align with the drum components and the timing of the song, requiring coordinated responses. This approach, which maintains spatial consistency and visual feedback, was also adopted in our MR application. Smash Drums additionally offer alternative visualization modes. One of them follows a paradigm inspired by Beat Saber, where the virtual drum set is not present, and floating drum-shaped targets approach the player in 3D space, requiring timed strikes as they reach specific positions. Another mode adopts a Guitar Hero-like approach, using a scrolling fretboard-style interface with vertical lanes representing drum components, where players must hit in precise rhythm as the notes descend.

In contrast, Paradiddle [13] is a true drumming simulator, offering a more realistic approach. It allows users to configure a custom drum set, connect MIDI devices such as electronic drum kits, and provides a range of tutorials for structured learning. This makes Paradiddle more aligned with our application’s educational focus. However, our approach differs significantly by integrating PocketDrum technology into an innovative way, leveraging the strengths of both motion-tracked air drumming and VR immersion to enhance the drumming experience beyond traditional setups. This combination also preserves tactile interaction with physical sticks, addressing one of the main limitations of fully virtual systems, where the lack of haptic feedback can reduce motor accuracy and disrupt the sense of embodiment [14].

In summary, prior VR and MR drumming applications have demonstrated the potential of immersive technologies for training and entertainment [3,4,7,11,12,13], including approaches based on avatar embodiment [5,7], vision-based gesture tracking [9], and controller-based interaction [11,12,13]. However, most of them rely on external sensors [6,10] or focus mainly on qualitative usability rather than systematic performance assessment [3,4,5,7]. To our knowledge, no existing system combines consumer-grade motion sensors with MR hand tracking in a unified framework, nor provides a dual evaluation including both technical performance (latency, jitter, sampling rate) and functional classification accuracy.

Accordingly, the central research question of this work is whether the fusion of inertial sensing and spatial hand-tracking data can effectively overcome the positional misclassification errors observed in inertial-only air-drumming systems.

## 3. Materials and Methods

### 3.1. Description of Technologies

#### 3.1.1. PocketDrum

**Figure 1:**
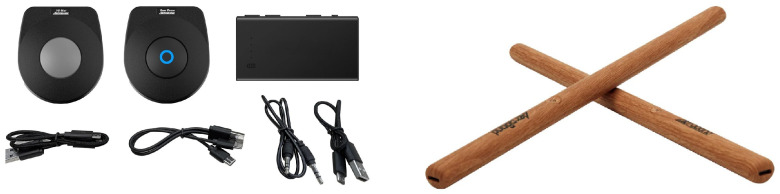
PocketDrum system including motion-sensitive drumsticks and foot sensors.

PocketDrum [15] is a pair of Bluetooth-enabled drumsticks equipped with embedded motion sensors that transmit strike events in MIDI format. Their portability and compatibility with multiple platforms make them suitable for MR-based drumming applications. A detailed description of their specifications is available in the manufacturer’s documentation. The full PocketDrum system, including drumsticks, foot sensors, and accessories, is shown in Figure 1.

#### 3.1.2. Meta Quest 3 Headset

The Meta Quest 3 [16] is a stand-alone MR headset that incorporates native hand tracking, enabling the 3D representation of the user’s hands without controllers. This allows natural interactions in both virtual and mixed environments. Its built-in cameras and computer vision algorithms provide real-time hand position data, although performance may degrade under low-light conditions or during very fast gestures. The system achieves an average fingertip positional error of approximately 1.7 cm [17], which is sufficient to distinguish between virtual drum elements typically separated by 15–20 cm. While the headset also includes physical controllers with higher precision, our application relies on the PocketDrum sticks combined with hand tracking to provide a natural drumming experience. Controllers are used only during the initial calibration step to position the virtual drum set.

### 3.2. Mixed Reality Application

**Figure 2:**
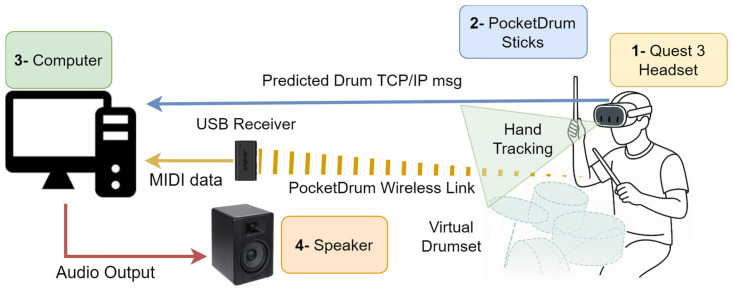
Physical layout of the MR drumming system showing the main components: 1—Meta Quest 3 headset, 2—PocketDrum sticks, 3—PC, and 4—speaker.

The system consists of four main physical components: the Meta Quest 3 headset (Meta Platforms, Inc., Menlo Park, CA, USA), the PocketDrum drumsticks (Aeroband, Shenzhen, China), a computer, and a high-powered speaker. Figure 2 illustrates the physical layout of these components in the MR drumming setup, showing how they are arranged to support real-time strike detection, sound playback, and synchronized visual feedback. The system integrates two complementary data streams: the PocketDrum drumsticks send strike events in MIDI format to the computer, while the Meta Quest 3 headset continuously transmits hand position data via TCP/IP. Upon receiving a strike, the computer uses both data sources to accurately determine which drum was hit and to generate the corresponding sound in real time. It also sends feedback information to the headset to update the visual scene.

#### 3.2.1. Design of the Mixed Reality Application

The goal of this MR application is to provide an immersive and effective drumming learning experience without the need for a physical drum set. We combine Meta Quest 3 hand tracking with PocketDrum strike detection to preserve stick-based haptics while enabling controller-free interaction in mixed reality. The design prioritizes natural movement execution, low-latency and reliable strike registration, and a spatial layout that matches real-world drum dimensions. To support different skill levels, the application integrates synchronized visual/audio guidance and configurable practice content. Calibration aligns the virtual kit to the player (see Section 3.2.3 for implementation details). The design was iterated with input from drummers, musicians, and educators to ensure pedagogical relevance.

#### 3.2.2. Description of the Mixed Reality Application

**Figure 3:**
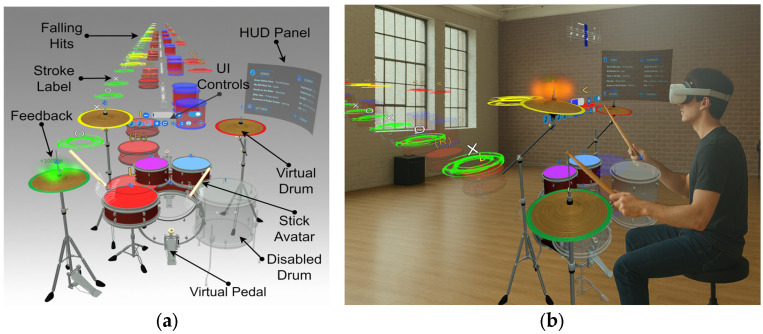
(**a**) User’s point-of-view showing the drumming interface with virtual elements and guidance cues (VR view). Passthrough (MR view) is used during calibration. (**b**) Composite image illustrating the external setup, including PocketDrum sticks and the Meta Quest 3 headset.

The user sees a virtual drum set composed of seven drums arranged in a standard configuration: Crash, Hi-hat, Hi-tom, Mid-tom, Ride, Snare, Floor-tom, and Bass, along with two-foot pedals for the Bass drum and Hi-hat. To play, the user holds the PocketDrum drumsticks and strikes in the air using the virtual drums as a reference. This interaction is illustrated in Figure 3, which shows both the user’s point-of-view and an external composite image of the system setup. To assist with learning, the application uses virtual discs synchronized with the music. These serve as visual guides to indicate the precise moment and location of each strike.

Before starting a song, the user must perform an initial calibration that aligns the virtual drum set with their real posture. This ensures that the interaction is both accurate and ergonomic, and that physical movements correspond properly to the virtual elements. Accurate calibration is essential to maintain a coherent correspondence between the user’s actions and the system’s responses. Previous studies have shown that sensorimotor mismatches in VR environments reduce the sense of agency and embodiment, (that is, the user’s feeling of controlling their own actions and of being physically present in the virtual body) hindering motor learning and performance [18].

Once calibration is complete, a graphical user interface appears beside the virtual drum set, leaving the front area unobstructed to maintain a clear view. This interface can be repositioned or minimized according to user preferences, enhancing the experience.

From the graphical user interface, users can choose from a variety of popular songs, basic rhythms, and drum rudiments organized by difficulty level. The selected rudiments follow standards defined by the Percussive Arts Society and include essential technical patterns such as rolls, flams, and paradiddles. These introductory exercises help users become familiar with the layout of the drums and understand how the virtual elements function, facilitating adaptation to the MR environment.

When a song or rhythm is selected, the application provides a short preparation period before starting, allowing the user to adjust their posture and drumstick position. Once the exercise begins, virtual discs appear and move from the background toward the corresponding drums, indicating the exact moment each one should be struck. Each disc represents a note in the piece and collides with the appropriate virtual drum in sync with the music. The speed of the discs is adjusted according to the tempo of the song, ensuring that the user has a clear visual reference to follow the sequence correctly. Discs can also be configured to match the shape and appearance of the corresponding drums, further improving visual identification.

Additionally, users can choose to play along with a backing track that plays the full song without drums, allowing them to integrate rhythmically with the other instruments and complement the visual information provided by the discs.

This moving disc system is the main visual support element in the application. It enables users, even those without prior music reading skills, to follow visual cues and perform songs while developing rhythmic coordination in an intuitive way.

**Figure 4:**
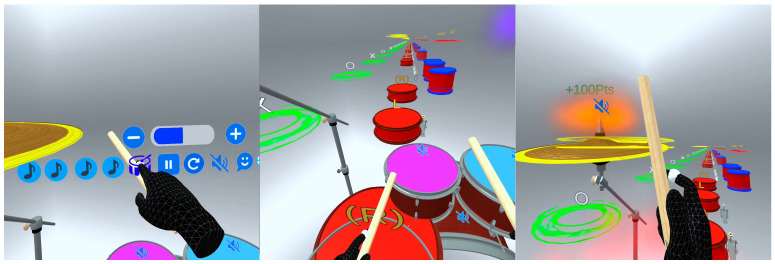
In-headset screenshots from the MR drumming application, captured using the Meta Quest Developer Hub. The images illustrate user interaction with the virtual drum kit, real-time visual cues, and the gameplay interface during rhythm exercises.

As a complement to the moving disc system, a linear representation of the sticking pattern is included, using auxiliary symbols such as “R” for right hand, “L” for left hand, “>” for accents, and “o”/“*” for open and closed Hi-hat. This visual guide is synchronized with the performance and dynamically highlights the current stroke, distinguishing past and upcoming hits using color codes. This feature is available for rudiments and for songs that include performance metadata, helping to reinforce the structural understanding of rhythmic patterns and optimize the user’s motor coordination. These visual elements (the moving discs and the dynamic sticking pattern) are illustrated in Figure 4, which shows in-headset screenshots of the MR drumming interface during rhythmic exercises. A demonstration video of the application is provided as Supplementary Material.

#### 3.2.3. Application Architecture and Technical Specifications

The mixed reality application was developed using Unity 2022.3.14f1 (LTS) together with the Meta XR Core SDK v74.2, which provided hand-tracking integration and real-time scene rendering on the Meta Quest 3 headset. Sensor data from the PocketDrum controllers were received through a custom TCP/IP protocol implemented in a C++ server application (Microsoft Visual Studio 2024, Windows 11). This program handled data acquisition, timestamping, and forwarding to the Unity client for synchronized visualization and interaction. The Unity project was built natively for Meta Quest 3 using the standard Android deployment pipeline and supported dynamic passthrough rendering controlled via script.

**Figure 5:**
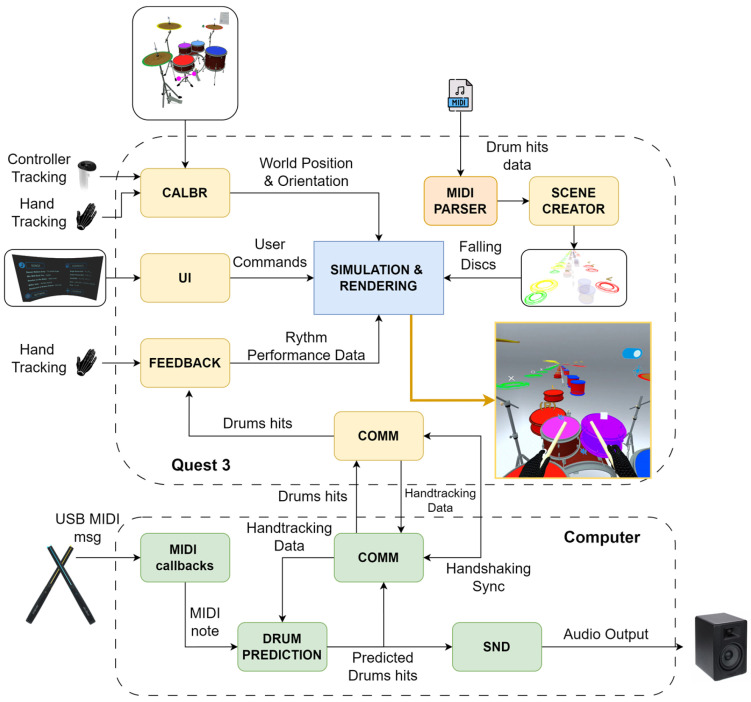
Overview of the system architecture, showing the interaction between the Meta Quest 3 headset and the computer for MR drumming application.

In terms of architecture, the system is composed of several modules distributed between the Meta Quest 3 headset and the computer, as shown in Figure 5.

##### Modules on Meta Quest 3

*Calibration:*

The calibration module aligns the virtual drum set with the user’s posture and physical configuration before playing begins. The system provides two manipulation handles, called gizmos, one for position and one for orientation, which allow the entire drum set to be moved and rotated within the virtual environment. These gizmos can be manipulated through direct interaction using controllers, enabling the user to intuitively configure the global placement of the set. Once the overall layout is defined, each virtual drum includes an individual anchor that can be used for fine adjustments, allowing the user to reposition specific drums as needed. During this calibration step, the Passthrough view is activated so that users remain aware of their physical environment while adjusting the virtual drums. Once the drums are in place, the foot sensors must be positioned manually, using the virtual pedals as a reference and leveraging Passthrough to visualize the real pedals superimposed in the environment.

Finally, the system includes a stick orientation adjustment mechanism, since hand tracking does not directly capture the drumstick itself. Orientation is inferred from hand, wrist, and forearm data, applying a standard grip model in which the thumb presses the stick against the fingers to ensure consistent and realistic alignment during performance.

*User Interface:*

The application allows users to interact with the interface in a natural way using the index finger. However, to prevent the virtual drumsticks from interfering with this interaction, the system includes a gesture-based switch: when both index fingers are brought together for one second, the drumsticks are hidden and a dedicated UI mode is activated, enabling direct interaction with menus and buttons. Repeating the gesture restores the drumsticks and returns the system to playing mode. This mechanism ensures smooth navigation without disrupting the drumming experience. To avoid accidental activations, a short dwell-time threshold is required to trigger the gesture, and interface elements are positioned in accessible locations without obstructing the view of the virtual drum set.

*Scene Creator:*

The Scene Creator module is responsible for generating the virtual discs that represent the notes to be played, based on the information contained in MIDI files. To do this, it extracts data from the drumming section, obtaining the start time and the assignment of each strike to a specific drum. However, since many songs include more than seven drums, the system reorganizes the original assignment, mapping the strikes to the drum configuration available in the application. This ensures that all notes are represented consistently without losing essential rhythmic information.

*Feedback:*

The Feedback module is responsible for processing information received from the computer to evaluate the accuracy of each strike. Whenever an impact is detected, it is compared with the expected sequence to determine whether it was correct or not, recording hits and misses in real time.

*Simulation and Rendering:*

The Simulation & Rendering module is responsible for animating the virtual discs generated by the Scene Creator, controlling their movement within the scene with precise timing.

*Communication:*

The Communication module manages the continuous exchange of data between the Meta Quest 3 headset and the PC via TCP/IP. It is responsible for receiving real-time hand position data and sending the processed strike information back to the headset, enabling immediate updates to the visual feedback. This module also ensures synchronization between devices, minimizing delays between the audio event and its visual representation in the scene.

Before interaction begins, the system performs a handshaking process to synchronize the internal clocks of the PC and the Meta Quest 3 headset. This involves calculating the Round-Trip Time, which is the time it takes for a data packet to travel from one device to the other and back. This value is used to properly align events generated on both devices and prevent timing discrepancies during execution.

##### Modules on the Computer

*Communication:*

This module serves as a counterpart to the communication module on Meta Quest 3, managing the message exchange protocol between the two devices. This includes handshaking to calculate Round-Trip Time, sending MIDI messages containing strike information, receiving hand tracking data, and handling other graphical user interface events.

*Drum Prediction:*

Once a MIDI message is received from PocketDrum, this module determines which drum was struck by using the most recent hand tracking data from Meta Quest 3. Although PocketDrum includes the corresponding MIDI note for the strike, this information is often unreliable, as it is not based on actual spatial positioning. Therefore, the system analyzes the hand’s trajectory just before impact and reassigns the strike to the most likely drum, combining PocketDrum’s fast detection with the spatial reference provided by Meta Quest 3. In practice, this module receives from the headset the continuously streamed 3D position and orientation of each drumstick tip, inferred within Meta Quest 3 from the hand, wrist, and forearm joints (as described in the *Calibration* section). The latest samples for each hand are stored in a short buffer, and a simple linear extrapolation is applied to predict the stick-tip position at the current timestamp, compensating for the ≈30 ms delay inherent to the 30 Hz hand-tracking rate. The predicted 3D position is then compared with the virtual drum layout, and the strike is assigned to the nearest drum element. This approach maintains accurate drum classification while minimizing the effect of tracking latency.

*Auxiliary Modules:*

The host-side MIDI and Sound Management modules handle strike input and sound generation, respectively. The former receives MIDI messages from PocketDrum and forwards them for processing, while the latter produces the corresponding drum sounds with minimal latency and maintains synchronization with the visual scene.

## 4. Experiment I: System Performance Analysis

Experiment I comprises three independent technical tests designed to assess the system’s core performance parameters: sampling rate, latency, and jitter. Each sub-experiment follows its own setup, procedure, and results section, reflecting the distinct aspects of temporal performance relevant to time-sensitive applications such as virtual drumming. The goal of these tests was to ensure that the hardware and software components performed reliably enough to support a real-time musical experience. The results of these evaluations informed key design decisions and helped identify potential limitations in system responsiveness.

All measurements were performed under a single optimized configuration to minimize external sources of variability such as ambient lighting, network conditions, and processing load. This approach allowed isolating the intrinsic performance of the integrated system. Although this controlled setup ensured reproducibility and internal validity, it represents a limitation, as results may vary under different environmental conditions or hardware configurations.

The experiments demonstrate that PocketDrum provides fast and stable strike detection, with low latency and a high sampling rate, allowing events to be recorded with great precision. However, lacking absolute positional information, it becomes unreliable to determine the exact location of each strike, limiting the accuracy of spatial assignment.

In contrast, Meta Quest 3’s hand tracking enables accurate three-dimensional tracking and facilitates integration with MR, offering better visual reference for the user. Nevertheless, it presents higher latency, greater variability in strike detection, and a lower sampling frequency, which can affect rhythmic accuracy during fast sequences.

Based on these results, a functional validation experiment was designed to evaluate how the combined system performs in realistic playing conditions, as described in the next section.

### 4.1. Sampling Rate

In this experiment, the sampling rate refers to the number of times per second the device captures motion data related to the user’s hand or drumstick position. An insufficient capture frequency may lead to missed strikes during fast passages, directly affecting rhythmic accuracy. Therefore, a sufficiently high sampling rate is essential to ensure reliable detection in high-speed sequences.

PocketDrum uses IMUs that record variations in acceleration and orientation in space. Although there is no official information regarding its exact sampling rate, it can be inferred to range between 200 and 500 Hz, in line with the energy constraints typical of such devices [17].

In contrast, Meta Quest 3’s hand tracking relies on computer vision: rather than directly measuring inertial parameters, the system analyzes images captured by the headset’s cameras to reconstruct the hands’ positions in real time. According to Meta’s documentation, the default hand-tracking mode operates at 30 Hz, while the recently introduced Fast Motion Mode (FMM), previously known as “High Frequency Hand Tracking,” raises the rate to 60 Hz. FMM is designed to improve tracking of fast movements, such as those common in fitness and rhythm applications, and is recommended only when significant tracking loss is observed with the default mode [19]. However, these values represent theoretical upper limits under ideal conditions; in practice, the effective sampling rate can be affected by factors such as processing load, ambient lighting, or speed of movement.

**Figure 6:**
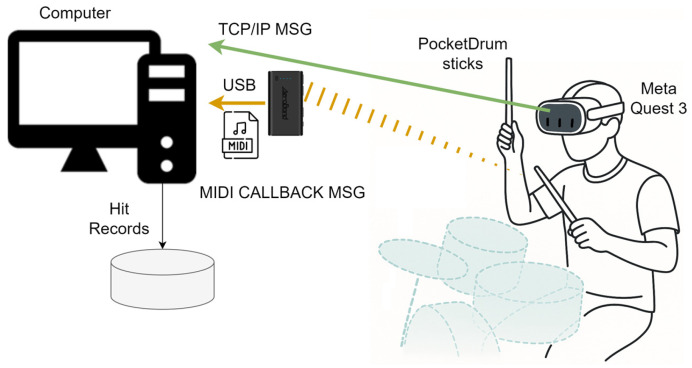
Experimental setup for strike detection analysis.

To assess the impact of sampling rate on real-time strike detection, we recorded sequences of repeated hits at various speeds, using both PocketDrum and Meta Quest 3 simultaneously. The PocketDrum drumsticks were connected via USB and sent MIDI signals, serving as the baseline reference, while Meta Quest 3 detected strikes through hand tracking and transmitted events via TCP/IP to a computer. In each trial, a professional drummer performed a short burst of continuous strikes at a steady speed, using either one hand or both hands in alternation, depending on the test. Each sequence lasted between 2 and 4 s, during which the system recorded the total number of real strikes (measured via PocketDrum), the number detected by Meta Quest 3, and the total duration of the trial. The procedure was repeated multiple times at increasing speed levels to span a wide range of strike densities. Detection rates were calculated by comparing Meta Quest 3’s event count against PocketDrum’s, disregarding latency. The experimental setup is illustrated in Figure 6.

Since PocketDrum operates with inertial sensors and a sampling rate significantly higher than the maximum speed achievable by a human user, its strike count was assumed to be nearly complete. This made it a suitable reference for evaluating Meta Quest 3’s performance under different conditions. Incremental speed tests were conducted using one and two hands, up to the point where the system began to miss events.

In default (30 Hz) mode, Meta Quest 3 began to miss strikes at around 6–7 hits per second, confirming that a limited capture rate can compromise detection in fast sequences. These results are summarized in Table 1. Individual trials were grouped according to similar average strike speeds, calculated by dividing the total number of PocketDrum strikes by the trial duration. For each speed level, detection rates were averaged across all corresponding trials to obtain representative values. When FMM (60 Hz) was enabled, performance improved significantly, reaching 12–13 hits per second without omissions, matching PocketDrum’s detection capability at the physical limit of human execution. Note that Table 1 reports only results from the default mode; in FMM Quest 3 sustained up to 12–13 hits/s without omissions, which we did not tabulate separately since detection remained perfect across the tested range.

For reference, a rock drummer playing with one hand in a regular groove can easily reach 3–5 hits per second. In extreme metal blast beats, alternating both hands, drummers can reach 10–15 hits per second. However, most typical rock grooves are played in a 4/4-time signature at tempos between 80 and 160 beats per minute (BPM), with each measure consisting of 4 beats. In standard patterns, the Hi-hat is struck at eighth-note subdivisions (twice per beat), while the Snare drum is played on beats 2 and 4, and the Bass drum (played with the foot) typically lands on beats 1 and 3. This results in approximately 10 hand strikes per measure, which corresponds to a range of about 3.3 to 6.7 hits per second when combining both hands. In the Notes column of Table 1, we include typical BPM values corresponding to the measured hits per second, along with a representative musical style, provided as a reference.

These results align with the sampling frequency range expected for inertial-sensor-based systems [15] and are consistent with values reported for commercial IMU devices used in similar VR/AR applications.

### 4.2. Latency

Latency is defined as the time elapsed between the user executing a strike and the system generating the corresponding response, either by sending a MIDI message or triggering sound playback.

**Figure 7:**
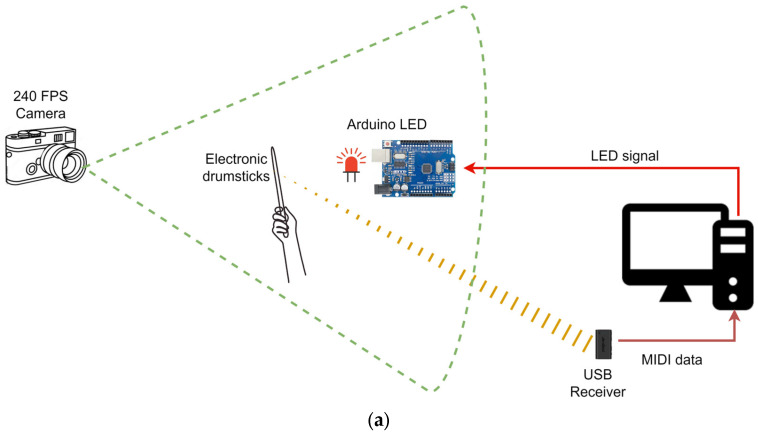

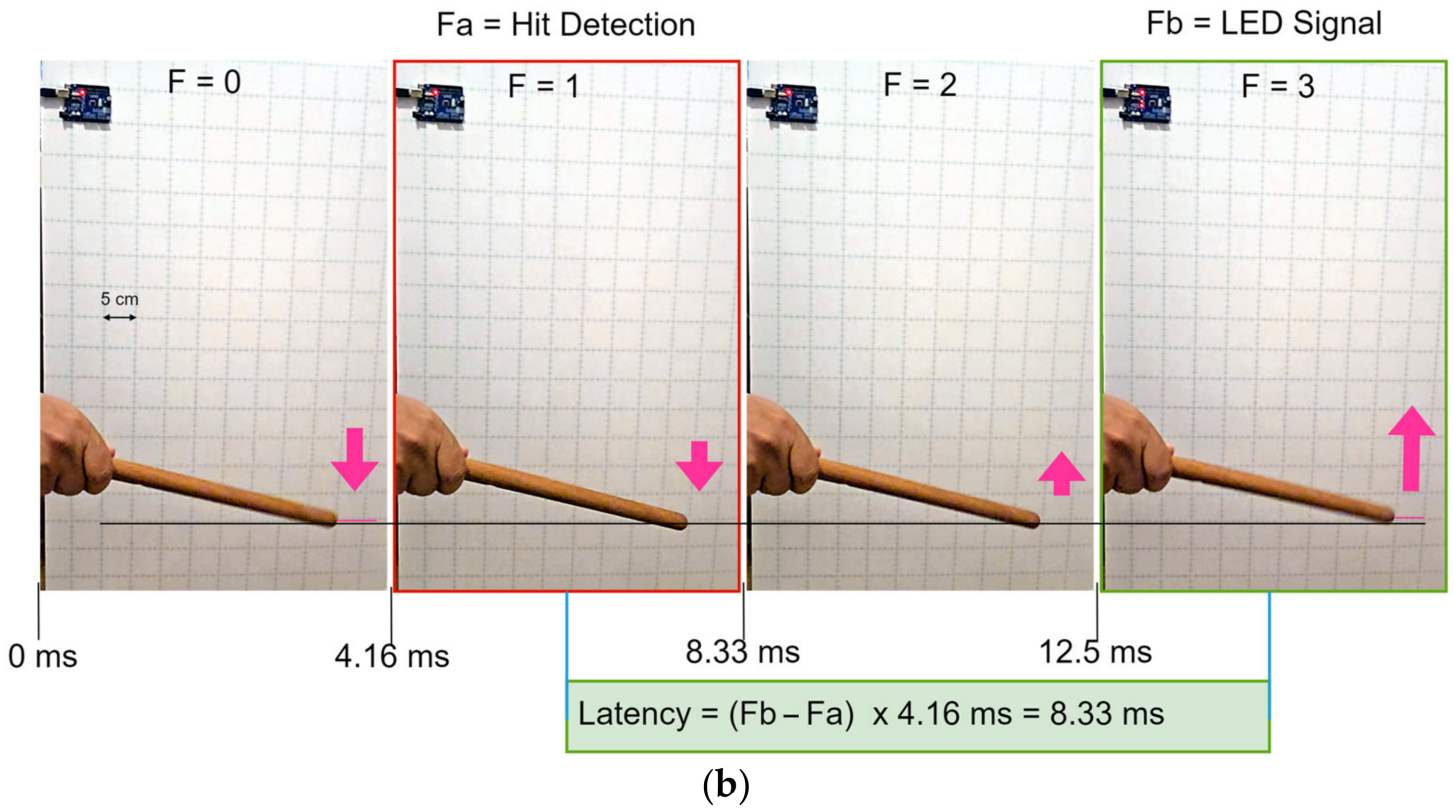
Experimental setup and timing analysis for latency measurement using PocketDrum. (**a**) Physical setup showing the connection between PocketDrum, the computer, the Arduino UNO board, and the LED used as a visual marker; (**b**) Frame-by-frame visualization of the stick’s motion and LED activation, used to determine latency between the physical strike and the system’s response.

According to PocketDrum’s official documentation, the drumsticks are advertised with a latency below 6 ms [15]. However, this value is not independently validated. To obtain an empirical estimate of the latency of the PocketDrum system, the drumsticks were connected via USB to a computer running a custom application that received MIDI strike events through the Windows callback mechanism. Upon receiving a MIDI message, the application immediately sent a command via USB to an Arduino UNO board, which activated its built-in LED on pin 13 for 15 ms. The entire setup was recorded using a high-speed camera at 240 FPS, allowing frame-by-frame analysis of both the physical strike motion and the exact moment the LED turned on. The experimental setup is illustrated in Figure 7a.

To quantify latency, the number of frames between the moment of visible strike impact (defined as the frame just before the direction change in the stick’s motion) and the first frame where the LED appeared illuminated was counted. Since PocketDrum can detect strikes slightly before the actual reversal point (due to the predictive nature of its inertial sensing) some latencies could appear negative. To normalize the results, all measured latencies were adjusted by adding 3 frames (the maximum early detection offset observed). This procedure yielded consistent and positive latency values across trials, capturing the complete detection chain from strike execution to MIDI generation, USB transmission, and LED activation. The experimental timeline is illustrated in Figure 7b.

The results showed an average latency of 8.17 ms (N = 100), with a 95% confidence interval ranging from 6.96 ms to 9.37 ms. This narrow range (±1.21 ms) indicates high temporal stability across trials. These values are fully compatible with the manufacturer’s claim of sub-6 ms strike detection latency, when accounting for the full signal chain—including MIDI message generation, USB transmission, operating system handling, and LED activation.

In the case of Meta Quest 3 hand tracking, there is no official information about its exact latency. However, according to a through-the-lens measurement, Quest 3 hand tracking exhibited an average offset of ≈31.3 ms between the real hand seen in Passthrough and the rendered virtual hand. Importantly, this value reflects only the relative desynchronization; when adding the ≈39 ms latency of the Passthrough pipeline itself, the total photon-to-hand latency in MR is around ≈70 ms [20], which is consistent with other informal measurements reported by developers and users.

More recently, a peer-reviewed study using a robotic testing framework reported that Quest 3 hand tracking latency can range from 14.4 ms to 220.5 ms, depending on hand posture, movement type, and distance from the headset [21]. This large variation confirms that, although Meta Quest 3 offers accurate spatial tracking, its temporal precision is significantly less stable. Given that PocketDrum exhibits much lower and more consistent latency (as confirmed in our measurements) no additional experiment using the Arduino LED setup was conducted for Meta Quest 3.

This latency range is fully compatible with the manufacturer’s claim of sub-6 ms detection [15] and comparable to latency values reported for commercial VR interaction systems [20].

### 4.3. Jitter

The motivation for this experiment arose from subjective reports collected during preliminary testing of our MR application based on hand tracking. Although the system correctly detected all strikes, some users reported difficulties in keeping the rhythm, describing a sense of temporal instability. This suggests that beyond average latency, the variability in the timing of event detection, known as jitter, may be affecting the experience.

In this experiment, jitter is defined as the fluctuation in the timing of events that should occur at regular intervals, and it is closely related to the sampling rate. Unlike constant latency, which may be perceptually tolerable, random variations in delay negatively impact musical synchronization. Latencies of around 10 ms or less are generally acceptable, yet adding jitter as small as ±3 ms significantly degrades perceived instrument quality [22].

Even in FMM (60 Hz), Meta Quest 3’s hand tracking imposes a theoretical limit on temporal stability. In the worst-case scenario, a strike could be detected up to 16 ms after it occurs, if it happens immediately after a sampling point and must wait for the next frame. In default mode (30 Hz), which is used in our application to preserve processing resources for other modules, this worst-case delay increases to approximately 33 ms. However, this is only an ideal boundary case, as in practice, other factors come into play that may increase variability, such as image processing on the headset, system load, or physics engine synchronization in Unity.

In contrast, PocketDrum uses IMU sensors with a high sampling rate, allowing for strike detection with very low temporal variability. As seen in the first experiment, latency remained consistently within a narrow range, between 6.96 ms to 9.37 ms. This stability allows PocketDrum to be considered a reliable reference system for evaluating jitter in Meta Quest 3, without requiring additional analysis of its own timing dispersion.

To evaluate this difference, we conducted an experiment in which a rhythmic sequence was recorded simultaneously with both systems. PocketDrum registered each strike as a MIDI event with microsecond-precision timestamps, while in Meta Quest 3, strikes were detected through Unity collisions and hand tracking events, recording their timestamps and sending them to the computer for analysis. For this test, the headset operated in FMM to ensure the best possible tracking performance. To correct potential clock differences between systems, the data was normalized by setting the first strike as t = 0, and deviations in detection times were compared.

**Figure 8:**
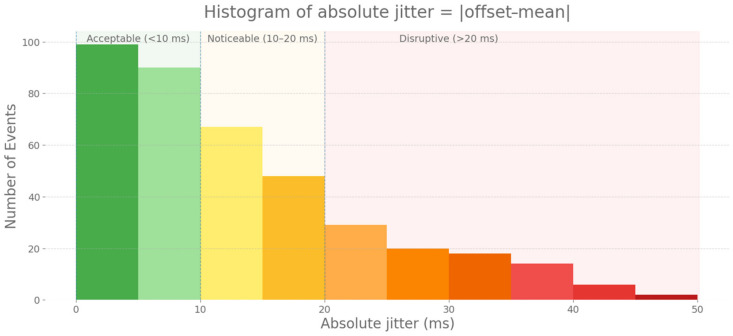
Histogram of absolute jitter (|offset − mean|) across all 393 paired events. Bars are color-coded by perceptual thresholds: green for <10 ms (acceptable), yellow–orange for 10–20 ms (noticeable), and red for >20 ms (disruptive).

Results indicated that the timing consistency of the Meta Quest 3 headset showed substantial variability in strike detection. Across 393 paired events, the average offset relative to PocketDrum was 1.38 ms. This constant component was removed to isolate variability, yielding a mean absolute jitter (mean|offset − mean|) of 13.27 ms. Most strikes fell within acceptable ranges, yet variability extended into perceptible deviations: 95% of events were within ±35.46 ms, 99% within ±41.05 ms, and the maximum reached 46.12 ms, which is sufficient to disrupt rhythmic stability even for untrained users. The distribution was heavy-tailed: many strikes were detected with small errors, but occasional large jitters introduced clear perceptual misalignments. These results are visualized in Figure 8, which shows the histogram of absolute jitter (|offset − mean|). Bars are colored according to perceptual thresholds: green for <10 ms (acceptable), yellow–orange for 10–20 ms (noticeable), and red for >20 ms (clearly disruptive). This explains the difficulties reported during preliminary testing: although tracking often appeared accurate, sporadic large jitters caused perceptible synchronization errors, making some strikes feel misaligned or inconsistent. The observed variability is within the range reported for vision-based tracking systems such as Meta Quest 3 [21], confirming that the temporal instability arises from the expected limits of optical hand-tracking technology.

## 5. Experiment II: Functional Validation Experiment

The objective of this experiment is to functionally validate the specific contribution of integrating hand position data from the Meta Quest 3 headset into the PocketDrum system. In particular, the experiment seeks to determine whether combining PocketDrum’s high-speed strike detection with spatial tracking from Meta Quest 3 fully eliminates the drum assignment errors that occur when using PocketDrum alone. The following subsections describe the procedure, dataset, logging method, evaluation criteria, and results of this experiment.

PocketDrum provides fast and consistent detection of strike timing but lacks positional awareness, often leading to misclassification of the target drum, especially in sequences involving adjacent or alternating hits. By adding the spatial data provided by Meta Quest 3 at the moment of each strike, the system can reassign the hit to the correct virtual drum based on the actual position of the user’s hand in space.

Although Meta Quest 3 hand tracking presents known temporal limitations, such as latency, jitter, and occasional missed detections, it offers highly accurate spatial tracking. Considering the average fingertip positional error of approximately 1.7 cm [12], and the fact that virtual drum elements are spaced 15–20 cm apart, the spatial resolution of Meta Quest 3 is more than sufficient to reliably distinguish between drums. Even under worst-case temporal delay (comprising 33.3 ms of frame sampling at 30 Hz and approximately 10 ms of network transmission latency), the total delay of 43.3 ms would require the hand to travel more than 30 cm during that interval in order to risk a spatial misclassification. This would imply a velocity of 7.5 m/s (27 km/h), which is far beyond the range of any realistic drumming motion.

In practice, sequences involving repeated hits on the same drum may result in missed tracking updates (as already discussed in Section 4.1 on sampling rates), but since the spatial position remains stable, these cases do not affect classification.

Therefore, if a strike is detected and position data is available, the system can confidently assign it to the correct drum. In this sense, hand tracking does not introduce classification noise but instead acts exclusively as a corrective mechanism for resolving the spatial ambiguities inherent to PocketDrum’s inertial detection.

Given these considerations, the MR system that combines PocketDrum’s high-frequency inertial strike detection with Meta Quest 3’s spatial hand tracking is established as the functional baseline for this study. All analyses of misclassification are thus based on deviations from this fused system’s output, which corrects the inherent limitations of using PocketDrum alone while maintaining robust classification consistency across all tested sequences.

### 5.1. Procedure

The experiment was conducted using the MR system in custom debug mode in default (30 HZ) hand tracking mode. In this configuration, each time a strike is detected by the PocketDrum sticks, the system logs detailed information about the event, including the original MIDI note reported by PocketDrum and the final drum assigned by the MR system based on real-time hand tracking from Meta Quest 3.

Before each test sequence, the PocketDrum sticks were calibrated according to the manufacturer’s guidelines. This involved holding the sticks horizontally at a 45-degree angle and briefly pressing the power button to initialize their reference orientation. This step was essential to ensure consistency across trials and minimize angular detection errors that could affect note classification.

The exercise set was designed to expose typical scenarios where PocketDrum is prone to misclassification. It included: (1) simple single-hand sequences, where no errors are expected; (2) spatially distributed patterns involving alternating drums with the same hand, where PocketDrum may assign incorrect targets due to its lack of absolute positioning; and (3) complex phrases and full song segments representative of real-world drumming.

In all exercises involving both hands (such as rudiments and full musical phrases) the left hand was assigned exclusively to the Snare drum. This design choice reflects standard drumming technique, where the non-dominant hand typically maintains the backbeat on the Snare. To avoid ambiguity and ensure valid comparisons, strikes performed by the left hand in these exercises were excluded from the evaluation dataset, since their outcome was predetermined by design and not subject to positional ambiguity. Notably, Snare strikes were included in the evaluation of single-hand exercises, where they were executed with the right hand and subject to the same criteria as other drums.

The dataset used for this experiment was collected from five participants with diverse profiles: two experienced drummers, one guitarist (considered a general user for this context), and two non-musicians with no formal training. Participants’ ages ranged from 19 to 53 years. None of the participants had prior experience using PocketDrum, ensuring that their performance was not biased by previous adaptation to the device’s limitations. The experiment was not designed to compare user performance or evaluate inter-subject variability. Instead, the focus was to expose the PocketDrum system to a broad range of realistic gestures and playing conditions. As such, not all users performed the same set of patterns. Some sequences, particularly the random tests, were unique to individual participants and not intended to be replicated. All collected events were aggregated into a single dataset for analysis, with no per-user segmentation. This approach allowed this study to focus on identifying systematic classification errors inherent to PocketDrum’s inertial-only detection under varied usage scenarios.

Each test sequence followed a standardized procedure: the PocketDrum sticks were calibrated, a rhythmic pattern was selected, and the participant repeated the sequence several times under controlled conditions. After each block of repetitions, a new pattern was chosen, and the sticks were recalibrated to ensure consistent interpretation of angular gestures. All test sessions were conducted in the same physical location under consistent artificial lighting. No session lasted more than 15 min, helping to minimize physical fatigue or cybersickness, and allowing participants to maintain consistent posture and performance throughout. Sessions were distributed over several weeks to accommodate participant availability and reduce fatigue-related variance.

Informal observations and user feedback were also collected during testing, including comments on interface design, visual elements, and usability. These inputs, while valuable for improving the application, are not part of the present analysis. Finally, it is important to emphasize that this experiment does not aim to evaluate user execution errors. The classification mismatches studied here result from the system’s misinterpretation of gestures, not from deviations in user performance. In particular, random test sequences were inherently non-repetitive and did not follow predefined sticking patterns, making execution “errors” conceptually inapplicable in those cases.

### 5.2. Logging and Data Structure

Each validated test trial generated a structured log containing a chronological sequence of strike events. For every strike detected by the MR system, the log recorded the original MIDI note reported by PocketDrum and the final drum assignment determined by the MR system using hand tracking data from Meta Quest 3.

The log format included the following fields for each event:

PD_Note: Drum indicated by PocketDrum’s MIDI message

MR_Assignment: Final drum assigned by the MR system based on hand position

This data structure enabled a direct comparison between PocketDrum’s original output, and the reassigned drum determined by the MR system. By identifying mismatches between the PD_Note and the MR_Assignment fields, it was possible to quantify how often the MR system corrected drum assignment errors that would have occurred using PocketDrum alone.

### 5.3. Evaluation Criteria

The evaluation focused on identifying and quantifying drum assignment errors made by PocketDrum and determining whether the MR system successfully corrected them using hand tracking. For each recorded strike, the system compared the original note reported by PocketDrum with the final assignment produced by the MR system. A mismatch between these two values was counted as a corrected assignment error.

To provide a more detailed analysis, the errors were examined across different dimensions: the specific rhythm sequence being performed, the virtual drum involved in the misassignment, and the hand used to execute the strike. This structure made it possible to detect patterns such as higher error rates in sequences involving fast alternation between adjacent drums, or asymmetries in error distribution between the dominant and non-dominant hand.

The primary metric for evaluation was the percentage of PocketDrum assignment errors that were corrected by the MR system. This allowed for a direct assessment of the effectiveness of the integrated positional tracking in resolving spatial ambiguities inherent to PocketDrum alone.

### 5.4. Results

A total of 2435 strike events were recorded across 35 rhythm sequences, including 5 rudiments, 23 short patterns, and 7 musical phrases or songs. Each strike was logged with both the original note reported by PocketDrum and the final drum assigned by the MR application based on hand tracking data. Of these strikes, 465 cases (19.1%, 95% CI: 17.4%, 20.8%) showed a mismatch between the original PocketDrum MIDI note and the drum assigned by the MR system, indicating an initial spatial misclassification that was corrected using positional tracking.

When results were analyzed by group, short single-strike patterns showed a relatively low error rate of 6.4%, and rudiments an even lower rate of 5.2%. In contrast, musical phrases and songs accounted for most mismatches, with a combined error rate of 33.7%.

When analyzing results by individual drum, error rates varied considerably. The highest mismatch percentages were observed on Crash (41.2%), Hi-tom (28.0%), and Mid-tom (26.0%). In contrast, lower error rates were recorded on the Snare (8.1%) and Ride (3.2%). The Snare showed consistent accuracy across all sequences, reflecting its stable performance under the test conditions.

When analyzing the performance across individual sticking sequences, substantial variation in mismatch rates was observed. For example, the S–MT pattern (Snare + Mid-tom) showed an error rate of 29.5%, while others like S–S–HT (Snare + Snare + Hi-tom) resulted in 0% mismatches. These differences indicate that the accuracy of drum assignment varies notably depending on the specific drum combinations used in each sequence. Table 2 summarizes the results. The data collected were analyzed descriptively (mean, standard deviation, and overall classification accuracy) to identify systematic patterns rather than to perform inferential statistical testing.

**Figure 9:**
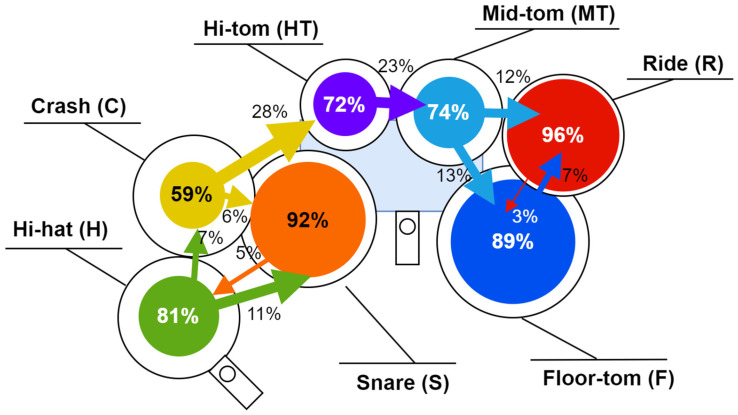
Spatial error mapping of drum classification. Each circle represents a drum in its real-world layout, with the area proportional to the percentage of correctly classified strikes. Colored arrows indicate misclassifications: their direction points to the incorrectly detected drum, and their thickness corresponds to the frequency of confusion.

To better understand common misclassification patterns, we analyzed the frequency with which each drum originally detected by PocketDrum was reassigned by the MR system. Figure 9 presents a spatial error map that visually summarizes these corrections, showing the most frequent confusion patterns between virtual drums.

Results confirm that Mid-tom was the most frequently misclassified element, followed by Hi-hat and Crash. Mid-tom exhibited cross-over with nearly every other drum, suggesting poor angular distinctiveness. Hi-hat was often confused with Snare, while Crash was reassigned to Ride in a substantial number of cases. In contrast, Snare and Ride showed high assignment consistency, reinforcing their stability under the current setup.

### 5.5. Limitations of the Experiment

This experiment focused exclusively on evaluating the functional behavior of the PocketDrum system in terms of its ability to correctly classify air drumming gestures without positional tracking. While the dataset included five participants with varied backgrounds and ages (19–53 years), the experiment was not designed to analyze inter-user variability or compare performance across participant profiles. Instead, all data were aggregated to identify systematic classification errors inherent to the device’s inertial-only detection method.

The experimental design did not include a formal assessment of user experience, perceived realism, or learning outcomes. Although informal feedback was collected regarding visual elements and usability, such comments were not analyzed in the present study. Additionally, no external motion capture system was used to independently verify classification accuracy; instead, the combined PocketDrum + Meta Quest 3 system was adopted as the internal functional baseline, based on its high spatial resolution and consistency, as discussed in Section 4.1 and Section 5.1.

Lastly, although the test protocol included stick recalibration before each sequence and limited session duration to prevent fatigue or cybersickness, the overall dataset remains limited in size. Additionally, the number of participants was limited, as the purpose of this experiment was to evaluate system performance and functional behavior rather than to conduct a statistical user analysis. Future research should consider expanding the number of participants, exploring novice learning curves, and incorporating structured user feedback and pedagogical evaluation metrics.

## 6. Discussion

This work presents, to our knowledge, the first MR drumming system that combines inertial sensing from PocketDrum with real-time hand tracking from Meta Quest 3 to enable spatially aware, contactless percussion practice. From a cognitive perspective, the integration of PocketDrum’s inertial sensing with Meta Quest 3’s spatial tracking can be interpreted as a technological embodiment of anticipatory musical cognition. Keller [1] describes how musicians rely on internal simulations of upcoming actions and sounds to maintain temporal accuracy and expressive control. Our MR environment externalizes this process: by providing real-time audiovisual feedback aligned with the user’s gestures, it reinforces the neural coupling between action and perception, potentially facilitating the same predictive mechanisms that support expert performance. This alignment between embodied interaction and anticipatory feedback highlights the pedagogical potential of sensor-fusion MR systems for rhythmic training. In contrast, previous applications in music education have instrument-centered visualization methods, for example by overlaying augmented guidance onto physical instruments such as a piano to support interpretation and coordination [23,24]. Drumming, however, poses a different set of challenges. Unlike a piano, it requires rapid, large-scale arm movements, precise timing, and spatial differentiation across multiple virtual targets. Several VR and MR systems have attempted to address this through various approaches, using avatars and embodiment to enhance expressiveness [5], AI-assisted training via virtual co-embodiment [7], or vision-based gesture detection without dedicated hardware [9]. Some commercial applications such as Smash Drums [11] and Drums Rock [12] favor game-oriented paradigms with abstract cues, while Paradiddle [13] offers a more realistic simulation with support for MIDI devices.

None of these solutions, however, integrate high-frequency inertial sensing with 3D hand tracking in real time for robust air-drumming classification. The system presented here introduces a hybrid sensing architecture that compensates for the limitations of each modality and enables accurate, real-time interaction without physical drum surfaces. This represents a novel contribution to the design of sensor-based musical systems, particularly in immersive, non-tactile environments. By combining the temporal precision of the inertial sensors with the spatial accuracy of hand tracking, the proposed configuration ensures gesture-to-sound alignment and temporal coherence, which are key requirements for rhythmic learning and musical feedback. Furthermore, the system provides a dual-level evaluation encompassing both technical (latency, jitter, sampling rate) and functional (classification accuracy) performance, which further differentiates it from previous VR or AR drumming applications.

The first experiment characterized the technical performance of the two sensing modalities independently, measuring latency, sampling rate, and jitter under controlled conditions. PocketDrum demonstrated low-latency strike detection (<10 ms), high temporal stability, and a sampling rate well above the upper limit of human drumming speed (estimated between 200–500 Hz). These properties make it highly reliable for capturing rhythmic timing. Meta Quest 3 hand tracking, on the other hand, offered precise spatial tracking with sub-centimeter resolution but showed greater variability in strike timing and occasional omissions due to its 30 Hz sampling rate. These measurements highlighted the individual strengths and limitations of each system and motivated the combined architecture used in the application.

The second experiment focused on the functional performance of the system in realistic musical scenarios. Strike sequences were grouped into rudiments, short patterns, and full musical phrases, revealing clear differences in error rates. Simple and repetitive patterns exhibited minimal spatial mismatches, while complex phrases and full songs were substantially more error prone. Similarly, some sticking combinations, such as alternating between Snare and Mid-tom, were notably more susceptible to misclassification than others involving fixed or symmetric gestures. At the instrument level, Crash and upper toms consistently showed higher mismatch rates, while Snare and Ride remained more stable. These results confirm that both gesture complexity and drum layout have a strong influence on classification reliability.

These patterns help explain the disproportionately high error rates observed in freeform musical exercises. Unlike short patterns or rudiments, these sequences span longer durations, include a wider variety of drums, and involve greater variation in posture and arm angle as the user moves across the virtual set. In the absence of spatial feedback, this variability leads to inconsistencies in gesture orientation, especially in combinations like Snare + Mid-tom, where similar angles can produce ambiguous signals.

In controlled sequences, users seem to adapt through repetition and maintain stable positions; but in longer or more exploratory contexts, the system’s reliance on angular estimation alone proves insufficient. The integration with Meta Quest 3 hand tracking effectively corrects many of these errors in real time, but the user remains unaware of these adjustments, which may limit their ability to detect and improve inaccuracies in their own technique.

It is worth noting that when PocketDrum is used in isolation, without visual feedback or positional correction, users often develop compensatory strategies over time. Through repetition, they learn to stabilize their gestures and adopt consistent angles for each drum, gradually reducing the rate of misclassification. Although this process requires more effort and adaptation, it demonstrates the user’s capacity to internalize the system’s limitations and achieve functional control with practice.

This two-stage evaluation (combining technical measurement with functional validation) provides strong support for the design of the MR drumming system. It shows that sensor fusion not only compensates for the weaknesses of each modality but also enables robust, real-time interaction suitable for musical training. The results validate the use of the combined system as a functional baseline for comparative analysis and demonstrate a practical approach to system validation in contexts where external ground truth is difficult to obtain.

Informal feedback from expert drummers, other musicians, and regular users who tested the application during development sessions further reinforced the technical findings. In total, over 10 users explored the MR system in non-standardized trials and reported a smooth and natural playing experience, with no perceptible lag and high comfort using the system. The visual indicators were consistently highlighted as helpful in maintaining orientation and improving coordination. Although these observations were not formally measured or included in the controlled experiments, they suggest that the system is well suited for immersive musical interaction.

Future work should expand this evaluation framework to include a more diverse user population, including students and novice musicians. Standardized subjective instruments such as the User Experience Questionnaire (UEQ) or System Usability Scale (SUS) could provide valuable insight into the perceived ease of use, engagement, and pedagogical value of the MR application. Longitudinal studies may also reveal how repeated use affects skill acquisition and retention compared to traditional practice methods.

Overall, the findings underscore the value of combining high-frequency inertial sensing with spatial tracking in musical applications. As hardware capabilities continue to advance, systems like the one described here may enable new forms of immersive, expressive, and portable music learning, practice, and performance.

## 7. Conclusions

This work presented a MR drumming system that combines PocketDrum and Meta Quest 3 hand tracking to enable accurate and immersive drum practice without the need for physical instruments. Through a series of technical evaluations and a functional validation experiment with participants of varied backgrounds, this study demonstrated that the integration of inertial and spatial data significantly improves strike detection accuracy compared to using either technology alone. PocketDrum alone was prone to misclassifying drum targets due to the absence of positional awareness, while Meta Quest 3 hand tracking occasionally missed strikes due to sampling rate limitations. Informal feedback from participants confirmed that the system was comfortable to use and that the visual guidance was especially helpful for maintaining orientation and improving coordination. The color Passthrough was considered useful during the calibration stage, but it was not required during musical practice.

These findings support the feasibility of using MR/VR systems for rhythm training and open the door to future developments in immersive musical learning. Further research will be directed toward expanding the evaluation to a broader user base, incorporating perceptual and pedagogical outcomes, and exploring adaptive or predictive algorithms to further improve responsiveness in fast or complex musical passages.

## Figures and Tables

**Table 1:** Detection rate of Meta Quest 3 hand tracking in default mode (30 Hz), under different strike speeds. Values represent the percentage of correctly detected strikes (mean ± 95% confidence interval (CI)) for grouped trials with similar average hit rates (hits/s). The “Notes” column provides typical musical tempo references corresponding to each strike speed.

Hands Used	Avg. Speed (Hits/s)	Detection Rates (95% CI)	Notes
One hand	1.6	100%, N = 342	Average user
	2.3	[98.6%; 100%], N = 210	
	3.6	[95.6%; 98.6%], N = 480	Drummer
	4.5	[93.6%; 98.1%], N = 313	
	5.9	[94.3%; 97.4%], N = 651	
Both hands	4.1	100%, N = 215	100 BPM—Ballad
	5.4	100%, N = 110	130 BPM—Pop Rock
	7.6	[97.3%; 99.1%], N = 221	180 BPM—Punk Rock
	9.3	[93.4%; 97.2%], N = 128	220 BPM—Thrash Metal
	11.1	[86.0%; 88.8%], N = 532	260 BPM—Extreme Metal
	12.9	[82.7%; 87.3%], N = 247	300 BPM—Death Metal

**Table 2:** Summary of PocketDrum assignment mismatches corrected by the MR system, aggregated by category. Values show total strikes (Hits), number of mismatches (Errors), and percentage of mismatches (%). The full per-sequence breakdown is provided in Appendix A (Table A1). The global proportion 95% CI is 17.4–20.8%.

Category	Hits	Errors	%
Short Patterns	1100	70	6.4%
Rudiments	194	10	5.2%
Songs	1141	385	33.7%
**Global Total**	**2435**	**465**	**19.1%**

## Data Availability

The data presented in this study are available upon reasonable request from the corresponding author.

## Supplementary Materials

The following supporting information can be downloaded at: Full video (DEMO): https://sensors-vrdrums-supplementary.s3.us-east-1.amazonaws.com/demo.mp4. Short version (Highlights): https://sensors-vrdrums-supplementary.s3.us-east-1.amazonaws.com/highlights.mp4.

## Appendix A

**Table A1:** Detailed per-sequence results of PocketDrum assignment mismatches corrected by the MR system. Values show total strikes (Hits), number of mismatches (Errors), and percentage of mismatches (%). Abbreviations: H = Hi-hat, S = Snare, F = Floor-tom, C = Crash, HT = Hi-tom, MT = Mid-tom, R = Ride.

Sequence	Drum							Total		
Sticking	H	S	F	C	HT	MT	R	Hits	E	%
**H-H-H-C-C-C**	18.5%			18.5%				54	10	18.5%
**H-S-H**	0.0%	5.9%						51	1	2.0%
**H-F-H**	2.9%		11.1%					53	3	5.7%
**H-F-R**	0.0%		0.0%				5.6%	54	1	1.9%
**H-C-H**	0.0%			10.0%				59	2	3.4%
**H-MT**	0.0%					15.0%		40	3	7.5%
**H-MT-H**	3.1%					6.3%		48	2	4.2%
**H-R**	19.0%						5.0%	41	5	12.2%
**H-R-H**	0.0%						0.0%	45	0	0.0%
**H-R-FT**	6.3%		6.3%				0.0%	48	2	4.2%
**S-S-H**	0.0%	5.3%						57	2	3.5%
**S-S-S-C-C-C**		0.0%		0.0%				36	0	0.0%
**S-S-C**		0.0%		21.4%				42	3	7.1%
**S-S-HT**		0.0%			0.0%			54	0	0.0%
**S-S-MT**		7.9%				0.0%		57	3	5.3%
**S-S-R**		12.5%					0.0%	48	4	8.3%
**S-C**		5.3%		10.5%				38	3	7.9%
**S-MT**		45.5%				13.6%		44	13	29.5%
**C-S**		6.7%		6.7%				30	2	6.7%
**HT-HT-MT**					5.3%	5.3%		57	3	5.3%
**HT-HT-R**					0.0%		0.0%	51	0	0.0%
**HT-MT**					8.0%	16.7%		49	6	12.2%
**MT-S**		4.5%				4.5%		44	2	4.5%
**Total**	**4.1%**	**8.1%**	**5.8%**	**11.5%**	**3.5%**	**9.2%**	**2.0%**	**1100**	**70**	**6.4%**
**Rudiments**										
**Double Stroke**							0.0%	44	0	0.0%
**Five Stroke**							2.8%	36	1	2.8%
**Flam**							12.5%	32	4	12.5%
**Paradiddle**							2.2%	45	1	2.2%
**Single Stroke**							10.8%	37	4	10.8%
**Total**							**5.2%**	**194**	**10**	**5.2%**
**Songs**										
**Rock-1**	19.9%		56.3%	100.0%		100.0%		222	78	35.1%
**Rock-2**	39.5%		21.4%	98.3%		50.0%		336	162	48.2%
**Random-1**	3.6%		5.0%	33.3%	100.0%	100.0%	0.0%	143	36	25.2%
**Random-2**	10.7%		0.0%	23.4%	81.8%	40.0%	0.0%	158	36	22.8%
**Random-3**	0.0%		0.0%	25.0%	16.7%	25.0%	0.0%	97	14	14.4%
**Random-4**	9.1%		0.0%	37.0%	52.6%	70.0%	5.0%	116	43	37.1%
**Random-5**	11.1%		0.0%	42.1%	100.0%	37.5%	0.0%	69	16	23.2%
**Total**	**27.4%**		**13.0%**	**54.6%**	**67.6%**	**54.1%**	**0.9%**	**1141**	**385**	**33.7%**
**Global Total**	**18.9%**	**8.1%**	**10.9%**	**41.2%**	**28.0%**	**26.0%**	**3.2%**	**2435**	**465**	**19.1%**
